# Variables and Mechanisms Affecting Electro-Membrane Extraction of Bio-Succinic Acid from Fermentation Broth

**DOI:** 10.3390/membranes12050542

**Published:** 2022-05-23

**Authors:** Alina Anamaria Malanca, Enrico Mancini, Mohamed Yusuf, Gabriel Kjær Khensir, Seyed Soheil Mansouri, Ioannis V. Skiadas, Hariklia N. Gavala, Manuel Pinelo

**Affiliations:** Department of Chemical and Biochemical Engineering, Technical University of Denmark, Søltofts Plads, Building 228A, 2800 Kgs. Lyngby, Denmark; alanma@kt.dtu.dk (A.A.M.); enrmini@kt.dtu.dk (E.M.); moe0207@hotmail.com (M.Y.); gabiih@live.dk (G.K.K.); seso@kt.dtu.dk (S.S.M.); ivsk@kt.dtu.dk (I.V.S.); hnga@kt.dtu.dk (H.N.G.)

**Keywords:** anionic exchange membrane, electrolytic cell, bio-succinic acid, separation and purification

## Abstract

The production of succinic acid from fermentation is a promising approach for obtaining building-block chemicals from renewable sources. However, the limited bio-succinic yield from fermentation and the complexity of purification has been making the bio-succinic acid production not competitive with petroleum-based succinic acid. Membrane electrolysis has been identified to be a promising technology in both production and separation stages of fermentation processes. This work focuses on identifying the key operational parameters affecting the performance of the electrolytic cell for separating succinic acid from fermentation broth through an anionic exchange membrane. Indeed, while efforts are mainly focused on studying the performance of an integrated fermenter-electrolytic cell system, a lack of understanding remains in how to tune the electrolytic cell and which main parameters are involved. The results show that a single electrolytic cell of operating volume 250 mL was able to extract up to 3 g L^−1^ h^−1^ of succinic acid. The production of OH^−^ ions by water electrolysis can act as a buffer for the fermenter and it could be tuned as a function of the extraction rate. Furthermore, as the complexity of the solution in terms of the quantity and composition of the ions increased, the energy required for the separation process decreased.

## 1. Introduction

A biorefinery is a promising alternative for overcoming the dependency on fossil fuels while at the same time addressing several contemporary challenges such as environmental problems, the depletion of petroleum resources, waste management, and political concerns [1]. Nowadays, worldwide efforts are being made to produce chemicals via biological routes. In this regard, succinic acid is widely recognized as a key building block for deriving both commodity and specialty chemicals [2]. Succinic acid is in fact one of the top 12 most used platform chemicals, being the precursor to 30 commercially valuable products, such as plasticizers, lubricants and solvents, pharmaceutical intermediates, and food and beverage additives [3]. The succinic acid market is projected to reach USD182.8 million by 2023, increasing at a CAGR of 6.8% from 2018 [4]. During recent years, four companies—Biosuccinium (former Reverdia), Succinity, BioAmber, and Myriant—have played a major role in the commercialization of succinic acid based on microbial fermentation [5]. However, despite efforts to make bio-succinic acid economically competitive, most of the succinic acid today is still produced from petrochemical derived sources [6], indicating that a higher market share of bio-succinic acid can only become possible by decreasing the total production costs. The major challenges lie in the availability of feedstock to secure a long-term biomass supply [7], the low productivity associated with fermentation [4], and the cost-intensive purification processes [8]. Regarding the feedstock challenge, intensive research has been done recently in terms of process synthesis and techno-economic analysis to find the best processing routes. One of the most important results of these studies was the identification of glycerol as the best feedstock for bio-succinic acid production [9]. A further attractive option would be the integration of the bioethanol production process with the bio-succinic acid production process. Indeed, glycerol and CO_2_ are by-products of bioethanol production, but they could also represent a secure long-time supply of feedstock for bio-succinic acid production [10]. The fermentation costs are mainly associated with product inhibition of the microorganisms and the cost of the base added to counteract acidification in the fermenter [11]. The separation of succinic acid from the fermentation broth is estimated to account for up to 80% of the total costs and thus represents the most important source of costs in bio-succinic acid production plants [8]. No specific method has been identified as the optimal for succinic acid separation and purification. However, in their review, Cheng [12] reported direct crystallization, precipitation, membrane separation, extraction, and chromatography as the major separation techniques for bio-succinic acid.

**Figure 1:**
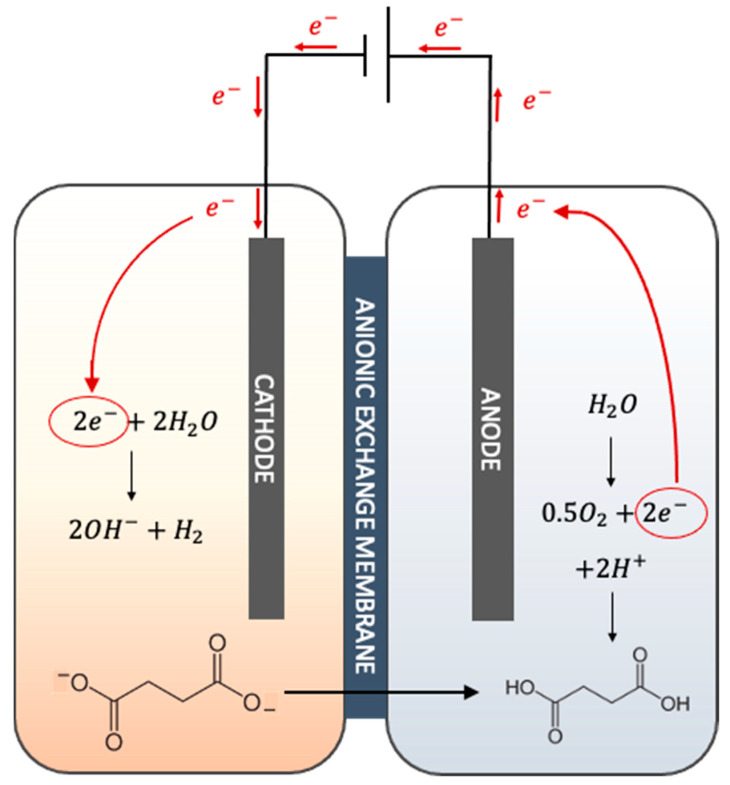
Schematic representation of succinic acid extraction in an electrolytic cell. The negatively charged succinate in the cathode chamber is driven through an anionic exchange membrane into the anode chamber where it is protonated to succinic acid. The applied voltage is the driving force that as a side effect brings about electrolysis of water to produce molecular hydrogen and hydroxide ions at the cathode and oxygen and protons at the anode side of the membrane.

Recently, membrane electrolysis has been shown to be a technology that could decrease the costs associated with both fermentation and downstream processing [13]. Membrane electrolysis is a novel electrochemical extraction technique in which electrodes are immersed in fermentation broth and upon the application of voltage drive the succinate, a charged acid anion, from the cathode compartment across an anion exchange membrane into the anode chamber. As a side effect, the electrolysis of water occurs in the electrolytic cell to generate molecular hydrogen and hydroxide at the cathode compartment and molecular oxygen and hydrogen ions at the anode chamber. A schematic representation of the electrolytic cell for succinic acid extraction is shown is Figure 1. From the fermentation point of view, a continuous extraction of the products would be advantageous because such a technique would reduce product inhibition. Furthermore, hydroxide ions generated during cathodic electrolysis can decrease the amount of alkali that needs to be added to the fermenter to counteract acidification. At the same time, the molecular hydrogen produced at the cathode can increase the production of reduced products by slowing down NAD^+^ regeneration via H_2_ production in many microorganisms, hence leading to improved conversion yields [14]. Pateraki [11] showed that in an integrated fed-batch *Basfia succiniciproducens* succinic acid fermentation with membrane electrolysis, productivity increased by 30%, while sodium hydroxide buffer usage was reduced by 33%. From the downstream point of view, the electrolytic cell has the potential to reduce the number of operational units. In fact, the membrane is not permeable to microbial cells and solids, which means that succinate extraction and concentration can be performed in a single operation. Moreover, the protons generated at the anode due to electrolysis can protonate the succinate to succinic acid, meaning that the addition of acid is not required for acidification [11].

While recent studies have given promising results regarding integrating membrane electrolysis with the fermenter, no studies have shown how to tune the electrolytic cell for such use. Our work thus focuses on characterizing an electrolytic cell for bio-succinic acid extraction by analyzing the different variables involved in the extraction rate. These variables include the voltage applied, the initial concentration and the distribution of organic acids in the electrolytic cell, the membrane area, the nature of the ions, and the batch versus the continuous setup. The experiments were performed in a 300 mL hand-made electrolytic cell in batch mode with a solution of pure succinic acid. The complexity of the solution was then increased to a mixture of acids, a synthetic broth of *A. succinogenes,* and a real fermentation broth of *A. succinogenes*. Finally, a continuous extraction of fermentation broth of *A. succinogenes* was simulated by recirculating the fermentation though a volume of 5 L of fermentation broth.

## 2. Theory

The electrolytic cell consists of two electrodes that behave as the active site for electron transfers for reactions that take place in an ionic solution that act as an electrolyte and can conduct electricity. Inside the electrolytic cell, two phenomena occur: electrolysis and the movement of charged ions towards the opposite electrode. The electrolysis of water produces molecular hydrogen and OH^−^ in the cathode chamber and oxygen and H^+^ at the anode site [15].

The equation describing the flux of ions under both a concentration gradient and an electric field is the Nernst–Planck equation:

(1) $$J_{i}=\nu \cdot C_{i}-{\;D}_{i}\cdot \frac{{\mathrm{dC}}_{i}}{\mathrm{dx}}-\frac{z_{i}\cdot F\cdot C_{i}\cdot D_{i}}{R\cdot T}\cdot \frac{d\phi }{\mathrm{dx}}$$

where $J_{i}$ is the ionic flux $\left({\mathrm{mol}\;m}^{-2}{\;h}^{-1}\right)$, ν is the convective velocity of the solvent $\left({m\;s}^{-1}\right),$ $D_{i}$ is the diffusion coefficient of ion $\left(m^{2}{\;s}^{-1}\right)$, F is the Faraday constant $\left({C\;\mathrm{mol}}^{-1}\right)$, R is the ideal gas constant $\left({J\;\mathrm{mol}}^{-1}K^{-1}\right)$, ϕ is the electric potential $\left({J\;K}^{-1}\right)$, T is the temperature (K), $C_{i}$ is the molar concentration, $z_{i}$ is the valence of ion, and x is a directional coordinate. The first term represents the convective transport of ions across the membrane, the second term is associated with the diffusion caused by the difference of concentration while the third term is associated with the diffusion caused by the voltage applied [16].

In two ionic solutions separated by an ionic perm-selective membrane as an anionic exchange membrane, a phenomenon called the Donnan effect (or the Gibbs–Donnan equilibrium) arises. Without a selective membrane between two solutions, the electric potential (φ) from the charged ions is in equilibrium (φ = 0). This is also called electroneutrality. When two solutions containing different concentrations of ions are separated by a fixed charged membrane, the two solutions are not in equilibrium and they create an electric potential gradient, either positive or negative [17]. This will result in the transfer of counter-ions through the membrane to reach an equilibrium between the solutions.

The relationship between current and voltage, the resistance, is an important factor to be investigated since the power needed for the system is directly proportional to the resistance, according to the formula:

(2) $$P=I^{2}R$$

where P is the power (W), I is the current (A) and R is the resistance (Ω). The resistance is related to both voltage and current according to Ohm’s law:

(3) $$R=\frac{V}{I}$$

where V is the voltage (V). The resistance is also directly proportional to the resistivity of the object, in this case the ionic solution, the electrode area, and the membrane, and it is indirectly proportional to the distance between the electrodes [18]. The conductivity of an ionic solution increases with the concentration of ions in the solution and with the temperature in step with an increase in the mobility of ions [19].

In this work the different terms of the Nernst–Plank equation, the Donnan effect, and the relationship between voltage and current were investigated experimentally to establish their influence on the extraction rate of succinic acid.

## 3. Materials

### 3.1. Experimental Organic Acid and Broth Solutions

**Figure 2:**
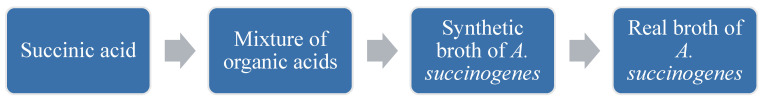
Simple schematic representation of four organic acid and broth solutions used in the experiments in order of time. The complexity of the solutions increases from a simple succinic acid solution to a real broth of *A. succinogenes*.

The experiments were conducted using increasing complexity solutions, from pure succinic acid to real broth of *A. succinogenes*. In total, four kinds of organic acid solutions were used with different components and concentrations. The first solution consisted of different concentrations of pure succinic acid. For the second solution, the complexity was increased to a mixture of succinic acid, formic acid, acetic acid and, pyruvic acid at a concentration ratio of 5:1:1:0.5, respectively. The reason behind this choice was that fermentation broths produced by *A. succinogenes* typically contain this kind of mixture and concentration of organic acids [20]. The third solution was similar to the previous solution but was modified by adding typical nutrients of a fermentation of *A. succinogenes,* as shown in Table 1. The fourth solution was a real fermentation broth of *A. succinogenes* based on Ferone [21]. However, the concentrations of succinic acid, formic acid, acetic acid, and pyruvic acid in this broth were adjusted to match their concentrations in the previous solutions in order to make the results comparable. A simple schematic representation of the solutions used for the experiments is shown is Figure 2.

Sodium hydroxide was used as a pH neutralizer for the solutions prior to the experiments to ensure that all the organic acids in the solutions were in ionic form and the solutions were electrically conductive. The above-mentioned chemicals used were obtained from Sigma Aldrich (Saint Louis, MO, USA).

### 3.2. Electrolytic Cell and Experimental Setup

**Figure 3:**
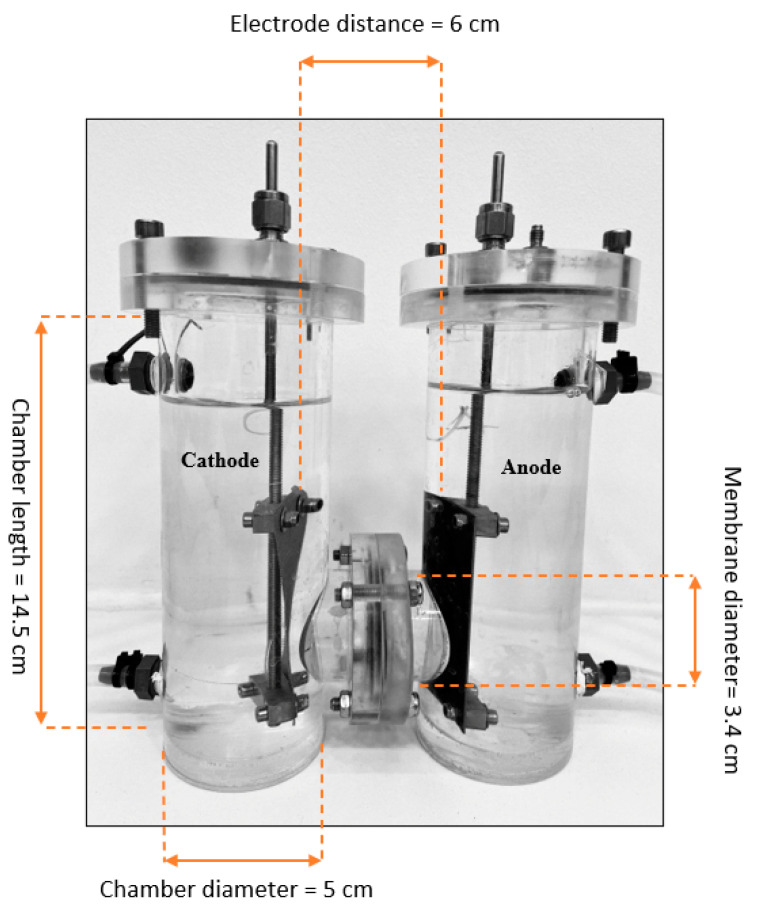
Front view of the electrolytic cell used for the experiments. The cell consisted of two 300 mL cylindrical chambers separated by a cylinder in which the anionic exchange membrane could be placed. The chambers contained the electrolytic solution into which the electrodes, an anode, and a cathode, respectively, were inserted at the distance of 6 cm.

The design of the cell was based on the work of Thygesen [22] and consisted of two cylindrical acrylic chambers of the same size connected with a circular tube where the anionic exchange membrane was installed, as showed in Figure 3. Each chamber had an internal diameter of 5 cm and a height of 14.5 cm and could accommodate a maximum volume of 300 mL. A custom-made stainless-steel cathode electrode (source: in-house workshop, Technical University of Denmark) and an iridium-coated titanium dioxide anode electrode from Magneto Special Anodes B.V (Schiedam, Netherlands) were used to activate the electrolytic reactions. Both the anode and cathode electrodes had an area of 7.8 × 3.8 cm^2^ and were placed at a distance of 6 cm from each other. The electrodes were attached to a power supplier EL302RT TRIPLE POWER SUPPLY from TTi. The anionic exchange membrane was a polystyrene membrane cross-linked with divinylbenzene from Membranes International inc. (Ringwood, NJ, USA) pre-treated accordingly, and the maximum membrane diameter used was 3.4 cm.

**Figure 4:**
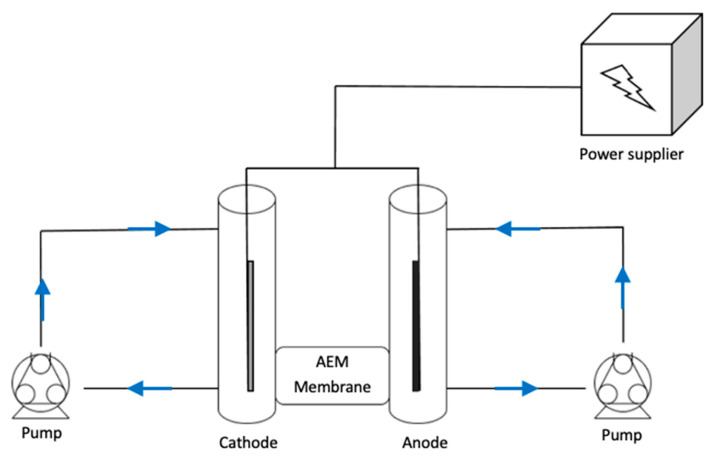
Illustration of the experimental setup of the electrolytic cell in batch mode. The organic acid or broth solution was recirculated between each chamber by peristatic pumps while the electrodes were attached to a power supply to create an electrical driving force from the cathode to the anode chamber. An anionic exchange membrane was inserted between the electrode chambers to ensure a perm-selective extraction of succinic acid and other anions in the solution.

**Figure 5:**
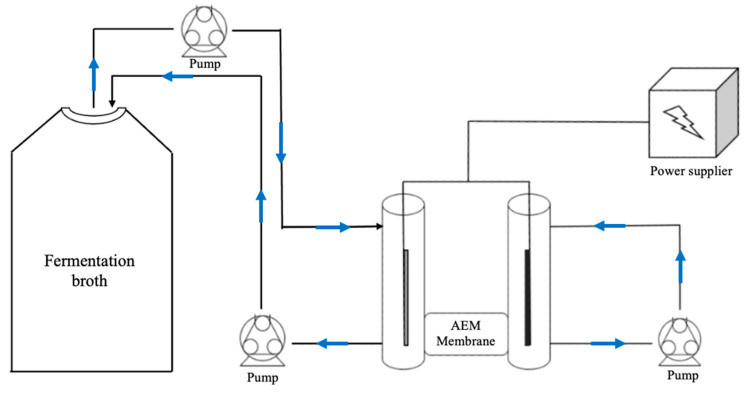
Illustration of the experimental setup of the electrolytic cell in continuous mode for fermentation broth of A. succinogenes. The solution was recirculated between the cathode chamber of the electrolytic cell and a 5L volume of fermentation broth, to simulate the coupling of an electrolytic cell with a fermenter. The anode chamber retained the same configuration as for batch mode.

The four types of solutions containing organic acids were used initially in batch mode. Each chamber of the electrolytic cell was filled with 250 mL of solution that was recirculated between the bottom and top part of each respective chamber using a peristaltic pump from Watson-Marlow (Ringsted, Denmark), model 523Du, with the liquid exiting the bottom section and entering the upper section of the chamber for mixing purposes, as shown in Figure 4. The experiments with real broth of *A. succinogenes* were performed both in batch and continuous mode. The continuous mode setup is illustrated in Figure 5. This setup was designed as far as possible to mimic coupling of the electrolytic cell with a fermentation tank. In practice, the solution in the cathode chamber was recirculated between a 5 L tank containing the fermentation broth of *A. succinogenes*, while the anode chamber contained 250 mL of solution as in batch mode. In this way the decrease in the concentration of succinic acid at the cathode was negligible because the succinic acid was extracted at the anode. In both setups, samples were taken from a sampling port attached to the recirculation tubes using single-use syringes.

## 4. Methods

The performance of the electrolytic cell was assessed based on multiple parameters. The key parameters were the extraction rate of succinic acid (g L^−1^ h^−1^) as a function of the current variation, concentration variation, ions composition and distribution, solution complexity, membrane area, and batch/continuous mode configuration. The experiments were performed as duplicates and the duration of each experiment was 3 h with sampling every hour. The first sample was taken at time = 0 and was not exposed to applied voltage. All the remaining samples were exposed to applied voltage during the experiment. For both anode and cathode chambers, the same solution was used. The reason for not using an inorganic anolyte, which could assist the Donna–Gibbs effect, was to avoid risking unwanted reactions and inserting a new variable. We arrived at this solution after demonstrating that the extraction rate was not improved by using a common anolyte such as sodium sulphate instead of succinic acid as anolyte. Table 2 shows the types of experiments performed with the different kinds of solutions.

### 4.1. Succinic Acid Solutions

#### 4.1.1. Effect of the Concentration in the Absence of Applied Voltage

The aim of this experiment was to assess whether the concentration gradient between the two chambers of the electrolytic cell has a relevant effect on the extraction rate of succinic acid in the absence of applied voltage, and also to determine the influence of the second term in the Nernst–Planck equation. The initial concentration of succinic acid was 50 g L^−1^ adjusted with sodium hydroxide to reach pH 7 at the cathode, and ultrapure water was present at the anode. However, the Nernst–Plank equation does not consider the Gibbs–Donnan effect, which can contribute to diffusion of the ions from one chamber to the other in the absence of electric driving forces. Actually, at the cathode at time 0 there is a solution of negative ions (succinate and OH^−^ from sodium hydroxide) that is locally neutral. If the succinate crosses the anionic exchange membrane to pass to the anode chamber, the cathode chamber becomes positively charged due to a lack of these negative charged ions. At this point a potential gradient is established across the membrane, with succinate in the anode chamber being attracted back to the cathode chamber. To demonstrate the effect of the Gibbs–Donnan effect, an experiment was performed in which initially the cathode chamber contained 50 g L^−1^ of succinic acid at pH 7 and the anode chamber contained a solution of sodium chloride instead of ultra-pure water. The concentration of sodium chloride was 24.76 g L^−1^ that corresponded to a molar concentration twice that of succinate because succinate carries a double negative charge. In this situation, the negative chlorine ions could substitute for succinate ions by crossing the anionic exchange membrane from the anode to the cathode chamber and theoretically maintain local charge neutrality.

#### 4.1.2. Variation in the Distribution of Ions

Two experiments were performed and compared to assess what initial distribution of ions inside the two chambers was necessary to maximize the extraction rate of succinic acid. In one experiment the initial concentration of succinic acid in the cathode chamber was 50 g L^−1^ while the initial concentration of succinic acid in the anode chamber was 5 g L^−1^. In the other experiment this distribution was reversed and the concentration of succinic acid in the anode chamber was more concentrated. The current was kept at 192 mA.

#### 4.1.3. Current Variation

This investigation aimed to assess extraction rate as a function of the current. The study consisted of five different experiments performed for 3 h with the same initial succinic acid concentration of 50 g L^−1^ in the cathode chamber and 5 g L^−1^ in the anode chamber. The potentiometer attached to the electrodes allows voltage to be controlled and current to be read, or the opposite way around. The authors chose to manipulate the current to keep it fixed and to read the voltage, which was slightly fluctuating during the experiment. This phenomenon might be due to the fact that the local concentration of ions changes continuously with time because the succinic acid is migrating and causes the conductivity of the solution to change. As a consequence, the solution resistance, to which the current and voltage are correlated, also changes. Another source of fluctuations could be change in temperature over time due to the exothermic oxidation reaction at the anode. In effect, the conductivity of an ionic solution increases with temperature. The currents at which the above-mentioned study was performed were 7 mA, 36mA, 96 mA, 192 mA, and 420 mA.

#### 4.1.4. Concentration Variation

In this part of the study, a comparison between different concentrations was performed at a fixed current of 36 mA, with 50 g L^−1^ pure succinic acid in the cathode chamber and 5 g L^−1^ pure succinic acid in the anode chamber, and 5 g L^−1^ in the cathode chamber and 0.5 g L^−1^ in the anode chamber. The aim of this experiment was to assess the importance of the initial concentration in the electrolytic cell for succinic acid extraction.

### 4.2. Mixed Acids Solution

#### 4.2.1. Organic Acids Variation

The solutions of mixed acids were composed of four types of organic acids, succinic acid 5 g L^−1^, formic acid 1 g L^−1^, acetic acid 1 g L^−1^, and pyruvic acid 0.5 g L^−1^. This mixture was similar to the composition of a real broth of *A. succinogenes* without the medium, based on a study by Lin [20]. The aim of this experiment was to investigate whether and how the addition of other monovalent ions changes the extraction rate of succinic acid in respect to the experiments conducted with pure succinic acid only (see Section 3.1). It was also important to gain knowledge on whether this configuration changes the voltage needed to maintain the same current density as in the pure succinic acid experiments. The experiments were conducted for 3 h at 36 mA. The composition of the synthetic broth is given in Table 3.

An experiment was performed with equimolar concentrations of the different organic acids to understand whether there is a difference in the extraction rate between the different organic acids. The aims of the experiment also included examining whether a divalent ion such as succinate has a higher extraction rate than a monovalent ion such as acetic acid, pyruvic acid, and formic acid. The experiment was performed with an initial concentration of 40 mmol L^−1^ of the 4 organic acids in the cathode chamber and 20 mmol L^−1^ in the anode chamber, which corresponded to 5 g L^−1^ of succinic acid in the cathode and 2.5 g L^−1^ of succinic acid in the anode at 36 mA.

#### 4.2.2. Variation of Membrane Area

In this experiment the diameter of the membrane was reduced from 3.4 cm to 2.4 cm, an approximate 30% decrease in diameter size. This was done by covering parts of the membrane with impermeable, non-conductive polystyrene (PP) plastic that blocked the passage of ions and solute. The intention was to observe whether the extraction rate of succinic acid decreased proportionally and what voltage was required to achieve the same current.

### 4.3. Synthetic Broth of A. succinogenes

#### Ion and Composition Variation

The synthetic broth of *A. succinogens* is a solution of mixed organic acids with the addition of the medium nutrients that constitute the typical synthetic fermentation broth of *A. succinogenes*. The aim of the experiment was to evaluate whether the addition of minerals and other compounds has a significant influence on the succinic acid extraction rate compared to the simpler solutions examined previously. The composition of the synthetic medium is given in Table 4, based on the study of Ferone [21].

### 4.4. Real Broth of A. succinogenes

For these experiments real fermentation broth of *A. Succinogenes* was used, based on the paper of Ferone [21]. The broth was first centrifuged to remove cells and solids and the composition was adjusted to have a concentration of succinic acid 5 g L^−1^, acetic acid 1 g L^−1^, formic acid 1 g L^−1^, and pyruvic acid 0.5 g L^−1^ to facilitate comparison with the previous experimental setups. The experiments were performed both in batch and continuous mode. For the continuous mode the solution in the cathode chamber was recirculated between a 5 L bottle of fermentation broth that had the same composition as that used in batch mode.

## 5. Results and Discussion

### 5.1. Effect of Concentration on Succinic Acid Extraction

The experiment with a 50 g L^−1^ initial concentration of succinic acid in the cathode chamber and ultra-pure water in the anode chamber aimed to assess the influence of the concentration gradient on succinic acid extraction in the absence of an applied voltage. At the same time the second term of the Nernst–Plank equation was investigated. The results showed that the concentration gradient is a very small driving force whose effect may be considered negligible within the scope of the experiment. In fact, no substantial amount of succinic acid was detected in the anode chamber at the end of the experiment, which is in accordance with the Nernst–Plank equation here:

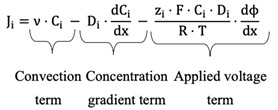
(4)

A brief comparison of the order of magnitude of the second term, which is associated with the concentration gradient, with the third term of the equation, which is associated with the diffusion of ions due to applied voltage, showed that the main driving force is associated with the voltage applied. For an initial succinic acid concentration of 50 g L^−1^ and an applied voltage of 30 V, the ration between the third and second is examined in the example below:

(5) $$\frac{\mathrm{Applied}\;\mathrm{voltage}\;\mathrm{term}}{\mathrm{Concentration}\;\mathrm{gradient}\;\mathrm{term}}=\frac{\frac{z_{i}\cdot F\cdot C_{i}\cdot D_{i}}{R\cdot T}\cdot \frac{d\phi }{\mathrm{dx}}}{D_{i}\cdot \frac{dC_{i}}{\mathrm{dx}}}=\frac{\frac{z_{i}\cdot F\cdot C_{i}\cdot D_{i}}{R\cdot T}\cdot \frac{\Delta \phi }{\Delta x}}{D_{i}\cdot \frac{{\Delta C}_{i}}{\Delta x}}=\frac{z_{i}\cdot F\cdot C_{i}\cdot \Delta \phi }{R\cdot T\cdot {\mathrm{\Delta C}}_{i}}=\;=\frac{2\cdot 96485\;C/\mathrm{mol}\cdot 423\;\mathrm{mol}/m^{3}\cdot 30\;J/C}{8.314\frac{J}{\mathrm{mol}\cdot K}\cdot 298\;K\cdot 423\;\mathrm{mol}/m^{3}}=2336.6$$

The above calculation shows that the contribution of the diffusion of ions due to a concentration gradient to ions transport is very low compared to electromigration, and that this outcome is valid for any concentration gradient chosen. The first term of the Nernst–Plank equation associated with convection was not investigated because the convection was not present in the system investigated.

**Figure 6:**
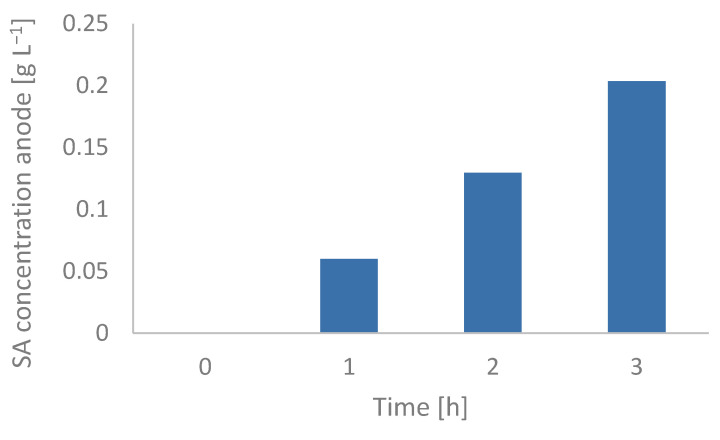
Measured concentration of succinic acid (SA) in the anode chamber during the concentration gradient experiment in the absence of applied voltage. Initial concentration in the cathode chamber was 50 g L^−1^ and initial concentration of NaCl was 24.75 g L^−1^, which corresponds to twice molar concentration with respect to succinic acid because succinate is double charged. The succinic acid was extracted in the anode chamber due to the Gibbs–Donnan effect.

However, the Gibbs–Donnan effect still seemed to be relevant in the absence of an applied voltage. In fact, repeating the previous experiment with a solution of NaCl in the anode chamber instead of ultra-pure water gave different results in terms of the extraction rate of succinic acid. Figure 6 shows that the concentration gradient is more relevant if there are negatively charged ions in both chambers of the cells, which can be exchanged across the membrane, and up to 0.2 g L^−1^ of succinic acid was extracted in 3 h. This was due to the fact that the succinic acid crossing the anionic exchange membrane from the cathode to the anode can be substituted by chloride ions, which are also negative, passing from the anode to the cathode, which enables the equilibrium to be maintained locally according to the Donnan law. This result did not occur for the experiments where ultra-pure water was present in the anode chamber. In that situation, the succinic acid crossing the membrane from the cathode to the anode could not be substituted by any other anion. As a result, a potential gradient developed across the membrane with a direction from the anode to the cathode, making the diffusion of succinate difficult.

**Figure 7:**
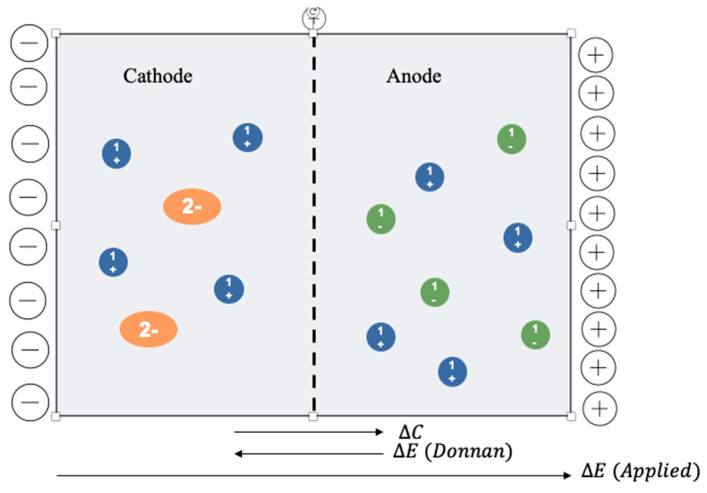
Schematic representation of the electrolytic cell and the main driving forces across the membrane: concentration gradient, Donnan potential, and external voltage applied. Sodium succinate (the orange double negatively charged circles) and sodium (the blue circles) are assumed to be present in the cathode chamber. Locally, the cathode chamber is neutral if no external voltage is applied. In the anode chamber sodium chloride is assumed to be present, with negatively charged chloride ions (the green circles). The anode is locally neutral in the absence of applied voltage.

The Donnan potential acts in different directions with respect to the applied voltage and can be considered to be almost negligible under applied external voltage. In fact, for the current experiments, the Donnan potential was significantly lower, in orders of magnitude of dozens of millivolts [17], when external voltage was applied in the range of 1.5–30 volts. A schematic representation of the main driving forces occurring in the electrolytic cell is shown in Figure 7.

The electrolytic reaction occurs inside the cell if an electrolyte is present in both the anode and cathode chambers. Both anolyte and catholyte solutions were prepared using succinic acid adjusted with sodium hydroxide to reach a pH of 7. However, it was not initially clear what distribution of ions was needed inside the two chambers to maximize the extraction rate of succinic acid. To determine the initial ion distribution inside the cell, an experiment was designed with an initial succinic acid concentration of 50 g L^−1^ in the cathode chamber and 5 g L^−1^ in the anode chamber, respectively. The results were then compared with another experiment where this distribution was reversed so that the anode chamber contained the higher succinic acid concentration. The results of these experiments show a decrease in the succinic acid extraction rate of around 38% when the anode chamber contained a higher succinic acid concentration than the cathode chamber. This outcome must be due to the fact that the cathode chamber solution, when more diluted, became less and less concentrated over time as ions were transported across the membrane to the anode chamber, consequently decreasing the conductivity of the solution in the cathode chamber and thus decreasing the whole extraction process. The results convinced us to perform the next experiments with the cathode chamber solution more concentrated than for the anode chamber.

Once we established that it is more convenient to operate the electrolytic cell with the cathode chamber more diluted than the anode chamber to increase the extraction rate of succinic acid, it was then necessary to establish the optimal anolyte and catholyte concentrations inside the cell. For this purpose, two experiments were designed and compared: one with an initial concentration of 5 g L^−1^ succinic acid in the cathode chamber and 0.5 g L^−1^ in the anode chamber, and one with 50 g L^−1^ succinic acid in the cathode chamber and 5 g L^−1^ in the anode chamber. The concentration in the cathode in the latter experiment was close to the maximum solubility of succinic acid in water, which is 58 g L^−1^ at 20 °C. The current was kept constant at 36 mA and the voltage was measured. 

**Figure 8:**
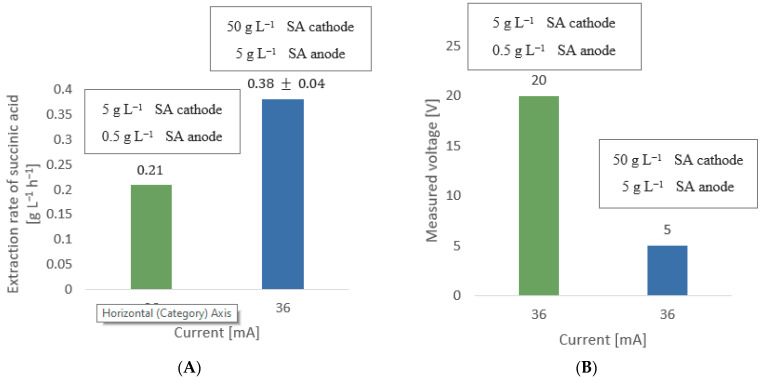
Two experiments are compared in terms of (**A**) extraction rate of succinic acid (SA) and (**B**) measured voltage as a function of initial concentration inside the electrolytic cell, both at a fixed current of 36 mA. The first experiment was performed with an initial concentration of 5 g L^−1^ succinic acid in the cathode chamber and 0.5 g L^−1^ of succinic acid in the anode chamber. This experiment was compared to a second experiment with 50 g L^−1^ of succinic acid in the cathode chamber and 5 g L^−1^ of succinic acid in the anode chamber. Note: SA = succinic acid.

The results, shown in Figure 8, demonstrate that the extraction rate of succinic acid was 81% higher for the most concentrated solution, which follows the Nernst–Plank equation that describes ionic flux as directly proportional to ion concentration. This means that in order to maximize the extraction rate of succinic acid, it is preferable to operate the cell at a high cathodic concentration. However, it should be noted that succinic acid fermentation does not usually produce such a high concentration of succinic acid. For example, Pateraki et al. [11] reported an extraction rate of 0.3 g L^−1^ h^−1^ in a fed-batch fermenter. Thus, in the case of a coupled continuous fermenter-electrolytic cell operation, an optimal residence time of succinic acid inside the fermenter must be defined before starting the extraction of succinic acid. With regard to the measured voltage for maintaining a current of 36 mA, an increase from 5 to 20 V was observed when passing from a more concentrated initial solution to a less concentrated solution. This observation strongly indicates that the conductivity of the solution increases when more ions are available. The result is a decrease in the resistance and consequently the voltage and power required. Therefore, working with a high initial ionic concentration would be beneficial both for the extraction rate of succinic acid and the energy consumption.

### 5.2. Extraction Rate of Succinic Acid as a Function of Current Variation

Since the concentration gradient and the Gibbs–Donnan effect were excluded as significant driving forces when a difference of voltage was applied, the electric potential gradient was examined by observing the extraction rate of succinic acid as a function of the applied current. The initial concentration of succinic acid was of 50 g L^−1^ in the cathode chamber and 5 g L^−1^ in the anode chamber, with the solution in the cathode chamber more diluted to maximize the succinic acid extraction rate, based on the previous results.

However, in comparing the experimental results with the theoretical Nernst–Plank, it is important to remember that the equation considers the voltage applied rather than the current. A relationship between voltage and current must therefore be established first for the system under investigation.

**Figure 9:**
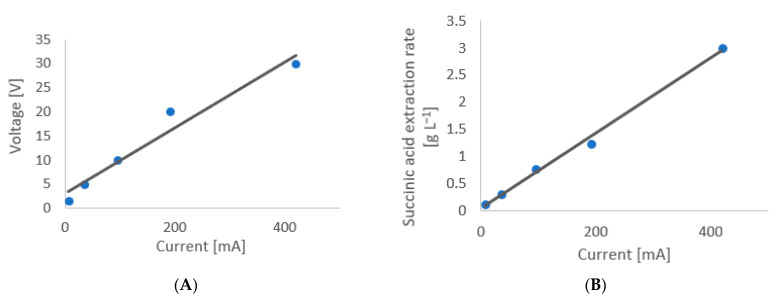
Relationship between (**A**) current and voltage for the examined system at the maximum voltage interval allowed by the potentiometer TTi and (**B**) extraction rate of succinic acid as a function of current during five different 3 h experiments. For both experiments the initial concentration of succinic acid in the cathode chamber was 50 g L^−1^ and the initial concentration in the anode chamber was of 5 g L^−1^.

Figure 9A shows that the voltage varies linearly with the current at the maximum interval allowed by the potentiometer, which indicates that the resistance is constant for the same solution. Figure 9B shows that the extraction rate changes with the change in current. This result is in accordance with the Nernst–Plank equation that states that flux across the membrane increases with an increase in potential gradient in a linear way. And since the relationship between current and voltage is linear, the extraction rate must also be linear with the voltage, in accordance with the Nernst–Plank law.

**Figure 10:**
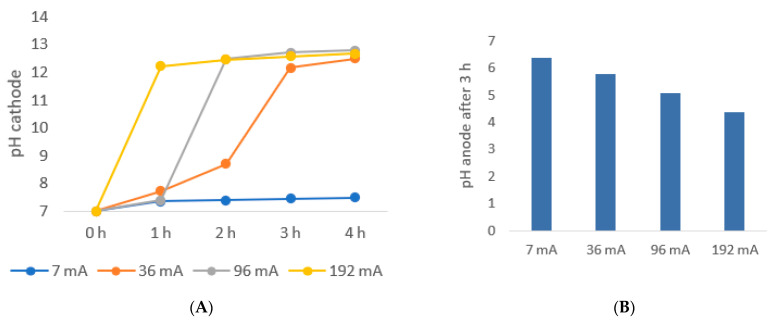
pH variation in the cathode (**A**) and anode (**B**) chambers as a function of time and current for a solution of succinic acid of initial concentration 50 g L^−1^ in the cathode and 5 g L−1 in the anode chambers. The variation at the anode side is the result of production of hydroxide ions from electrolysis while the variation in the anode chamber is the result of release of H^+^ ions from water electrolysis.

The electrolysis of water, which is a side effect of the applied voltage, occurs and produces molecular hydrogen and hydroxide in the cathode chamber, and molecular oxygen and hydrogen ion in the anode chamber. This effect causes the pH to rise in the cathode chamber and decrease in the anode chamber as a function of the current or voltage applied, as shown in Figure 10.

In both the anode and cathode chambers, the initial pH of the solution was seven. The pH at the cathode increased with increasing current applied, and the rate was faster at a higher current. The situation was particularly critical for the cathode chamber. Here pH increased greatly, but the chemical stability of the membrane lies within the range pH 1–10, and therefore subsequent experiments were performed at low current/voltage settings. The anode chamber pH was monitored only at the end of the experiments and was demonstrated as expected to decrease with the increasing applied current.

### 5.3. Mixed Organic Acid: Effect of Ion Valence on Acids Extraction Rate and the Pyruvic Acid Phenomenon

According to Ferone [21], in a typical fermentation of *Actinobacillus succinogenes,* the other main organic acids produced, apart from succinic acid, are acetic acid, formic acid, and pyruvic acid. Only succinic acid is divalent, while the other three organic acids are monovalent. According to the Nernst–Plank equation, this should result in a succinic acid extraction rate that is twice as high compared to the other monovalent acids, because ionic flux is directly proportional to the ion valence. An experiment with equimolar concentrations of the four organic acids resulted in an extraction rate of succinic acid at the anode chamber that was twice that of acetic acid, in perfect accordance with the Nernst–Planck equation. However, the extraction rate of formic acid in the anode chamber was approximately and significantly three times lower than the extraction rate of acetic acid, even though both acids are monovalent. A hypothesis that may explain this phenomenon is that formic acid in the electrolytic cell could react to form some other products not detected by the HPLC, since the total mass balance of formic acid was slightly decreased at the end of the process. The probable interaction between ions could also be a reason behind the different extraction rate of monovalent ions [23].

Regarding pyruvic acid, its mass balance was noted to not remain constant with time. In particular, the concentration in the cathode chamber decreased significantly with time, while the concentration in the anode chamber did not increase. This result indicates that pyruvic acid was lost or converted into another product at the cathode. No significant volume change was observed in the chambers throughout the experiment. A possible explanation is that pyruvate was converted to lactate in the presence of hydrogen according to the following reaction: 
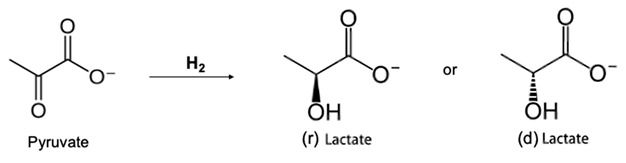


Actually, the HPLC showed a significant amount of lactic acid both in the cathode and in the anode chamber, which indicated that pyruvate was initially converted into lactate that in turn was extracted through the membrane because lactate is a negatively charged ion. This theory can be supported by literature [24] showing that pyruvate is converted into lactate in the presence of hydrogen, but this process does not seem to have been performed previously in an electrolytic cell. However, only 31% of the pyruvic acid that disappeared from the cathode chamber was converted into lactic acid, and the unaccounted-for pyruvic acid could have been reduced further, according to the literature, to propylene glycol or other diols [25].

Reducing the membrane area by 30% led to a decrease of 33% in the extraction rate of succinic acid in a mixed acid solution, while the voltage required to maintain the same current of 36 mA increased by 22%. This result suggests that a good strategy would be to use a larger membrane area that would therefore allow both a higher extraction rate and the use of a lower voltage to maintain the same current.

### 5.4. Comparison of the Extraction Rate and Energy Requirements of the Different Solutions

The extraction rate of the desired compound, succinic acid, was compared using different solutions and experimental setups as described in the Materials and Methods section. Specifically, 5 g L^−1^ of pure succinic acid was compared with a solution of mixed acids typically found in fermentation broth of *A. succinogenes* (5 g L^−1^ succinic acid, 1 g L^−1^ lactic acid, 1 g L^−1^ formic acid, and 0.5 g L^−1^ pyruvic acid) and to a synthetic broth of *A. succinogenes* containing the same components at the same concentrations to those in the mixed acid solution but including the addition of nutrients. The final comparison was performed using the real fermentation broth of *A. succinogenes* and with the operation of the electrolytic cell both in batch mode and in continuous mode. In continuous mode the solutions were recirculated between the cathode chamber of the electrolytic cell and a 5 L bottle containing the fermentation broth to mimic a coupled fermenter-electrolytic cell.

For the same current and initial concentration of succinic acid in the different solutions, the extraction rate of succinic acid was highest for the pure succinic acid solution. This is reasonable because the presence of the other organic acids and ions could induce a competition between the ions and decrease the extraction rate of the succinic acid itself while maintaining the same current. In fact, the lowest extraction rate was obtained for the synthetic broth of *A. succinogens* due to presence of many other ions in the solution. However, similar extraction rates were obtained for the batch and continuous modes. To highlight the competition of ions in the solution, the ratio between the total negative ions in the test solution (succinic acid solution and synthetic broth) and the ions of succinic acid extracted after 3 h, was calculated as a percentage. As expected, this ratio was found to decrease with increasing complexity of the solution due to the high probability of competition between ions. The real broth was not included in this comparison due to the uncertainty over total ions concentration and composition.

With regard to voltage and thus the energy requirements, the result indicates that the electrical resistance was lower due to the presence of more ions in solution, which is similar to what was observed when the initial concentration of succinic acid was more concentrated. The lower voltage in continuous mode compared to the batch mode probably reflects the recirculation of ions between the cathode chamber and the 5L tank, and therefore no decrease in ions when succinic acid is extracted in continuous mode compared to batch mode. Table 5 presents the results discussed.

## 6. Conclusions

The aim of this study was to assess the performance of an electrolytic cell with an anionic exchange membrane for succinic acid extraction and a range of parameters: the applied current, the initial ions concentration and ions distribution inside the electrolytic cell, the nature of the ions and the complexity of the initial solution, the membrane area, and batch versus continuous mode. We demonstrated that an electrolytic cell configured in this is able to extract succinic acid and that the extraction rate of succinic acid, given a constant current, decreases with increasing complexity of the solution, probably as a result of the competition between the ions in solution. However, the voltage needed to maintain the same current in the cell also decreases because the initial solution becomes more electrically conductive, meaning that less energy is required. There was no evidence that a continuous extraction would be more advantageous for extraction compared to a batch extraction. Other significant advantages of continuous extraction must be considered, however, such as the avoidance of product inhibition and the reduced use of buffer. Indeed, the experiments showed that a very high pH is eventually reached in the cathode chamber due to water electrolysis, which could be used as a potential way to control pH. Another advantage is the fact that the voltage required in the continuous mode was lower than in the batch mode. Future work could determine the optimal voltage needed to optimize the extraction rate and control the pH in a fermenter in an electrolytic cell-fermenter coupled system. If the intention is to reduce electrical resistance and power demands, a future cell must be designed with a larger membrane area and a smaller gap between the electrodes. Furthermore, membrane choice must also be carefully investigated to ensure that the membrane is chemically stable over the whole operational pH range.

Even though this study focused on succinic acid, the proposed methodology can possibly be extrapolated to other carboxylic acids produced by fermentation, such as acetic acid, and potentially to other ionic products.

## Figures and Tables

**Table 1:** Qualitative composition of the different types of organic acid solutions used in the experiments. The concentrations are not indicated in this table because they varied throughout the experiments.

Succinic Acid Solution	Mixed Acids Solution	Synthetic Broth of *A. succinogenes*	Real Broth of *A. succinogenes*
Succinic acid	Succinic acid	Succinic acid	Real fermentation broth
	Formic acid	Formic acid	
	Acetic acid	Acetic acid	
	Pyruvic acid	Pyruvic acid	
		NaCl	
		K_2_HPO_4_	
		NaH_2_PO_4_	
		MgCl_2_ × 6H_2_O	
		CaCl_2_ × 2H_2_O	

**Table 2:** Type of experiments performed with different organic acid and broth solutions.

Succinic Acid	Mixed Acids	Synthetic Broth of *A. succinogenes*	Real Broth of *A. succinogenes*
Osmosis study	Organic acids variation	Ion and composition variation	Batch versus continuous operation mode
Current variation	Membrane area variation		
Ion’s distribution variation			
Ion’s concentration variation			

**Table 3:** Composition of mixed acids solutions at the onset of the experiments in the anode and cathode chambers.

Chamber	Succinic Acid	Pyruvic Acid	Acetic Acid	Formic Acid
Cathode	5 g L^−1^	1 g L^−1^	1 g L^−1^	0.5 g L^−1^
Anode	0.5 g L^−1^	0.1 g L^−1^	0.1 g L^−1^	0.05 g L^−1^

**Table 4:** Initial composition and concentration of synthetic broth of A. succinogenes based on Ferone [21] at the onset of the experiment in the anode and cathode chambers.

Component	Cathode (g L^−1^)	Anode (g L^−1^)
Succinic acid	5	2.5
Formic acid	1	0.5
Acetic acid	1	0.5
Pyruvic acid	0.5	0.25
NaCl	0.3	0.15
K_2_HPO_4_	5	2.5
NaH_2_PO_4_	2.62	1.31
MgCl_2_ × 6H_2_O	0.067	0.034
CaCl_2_ × 2H_2_O	0.077	0.039
Glycerol	10	5

**Table 5:** Comparison of succinic acid extraction rate and voltage requirement of all experiments at a fixed current of 36 mA. The initial concentration of succinic acid (SA) in all solutions was of 5 g L^−1^. The measured variables are extraction rate of succinic acid and voltage.

Solution	Current (mA)	Cathode SA (g L^−1^)	Anode SA (g L^−1^)	Extraction Rate (g L^−1^ h^−1^)	% Extraction Rate of Ions of SA/Total Initial Ions	Voltage (V)
Succinic acid	36	5	2.5	0.25 ± 0.01	13.56%	8.54 ± 0.19
Mixed acids	36	5	2.5	0.23	7.3%	6.42 ± 0.21
Synthetic broth	36	5	2.5	0.15	2.62%	4.93 ± 0.11
Real broth (batch)	36	5	2.5	0.20 ± 0.01		6.52 ± 0.04
Real broth (continuous)	36	5	2.5	0.19		6.11 ± 0.01

## Data Availability

Data available upon request to the corresponding author.
